# Microbiome, Metabolome and Inflammatory Bowel Disease

**DOI:** 10.3390/microorganisms4020020

**Published:** 2016-06-15

**Authors:** Ishfaq Ahmed, Badal C. Roy, Salman A. Khan, Seth Septer, Shahid Umar

**Affiliations:** 1 Department of Surgery, University of Kansas Medical Center, 3901 Rainbow Blvd, 4028 Wahl Hall East, Kansas City, KS 66160, USA; iahmed@kumc.edu (I.A.); broy@kumc.edu (B.C.R.); 2 Department of Internal Medicine and Department of Pediatrics, University of Missouri, Kansas City, MO 64110, USA; khansal@umkc.edu (S.A.K.); ssepter@cmh.edu (S.S.)

**Keywords:** microbiome, metabolome, metabolites, dysbiosis, inflammation bowel diseases, prebiotics, probiotics

## Abstract

Inflammatory Bowel Disease (IBD) is a multifactorial disorder that conceptually occurs as a result of altered immune responses to commensal and/or pathogenic gut microbes in individuals most susceptible to the disease. During Crohn’s Disease (CD) or Ulcerative Colitis (UC), two components of the human IBD, distinct stages define the disease onset, severity, progression and remission. Epigenetic, environmental (microbiome, metabolome) and nutritional factors are important in IBD pathogenesis. While the dysbiotic microbiota has been proposed to play a role in disease pathogenesis, the data on IBD and diet are still less convincing. Nonetheless, studies are ongoing to examine the effect of pre/probiotics and/or FODMAP reduced diets on both the gut microbiome and its metabolome in an effort to define the healthy diet in patients with IBD. Knowledge of a unique metabolomic fingerprint in IBD could be useful for diagnosis, treatment and detection of disease pathogenesis.

## 1. Defining Inflammatory Bowel Disease

Inflammatory bowel disease (IBD) is a prolonged and relapsing inflammation of all or part of the gastrointestinal (GI) tract. Inflammation impairs the functioning of affected GI organs leading to abdominal pain, persistent diarrhea, cramping, weight loss, rectal bleeding, and fatigue *etc*. IBD may result in compromised quality and expectancy of life with increased risk for colorectal cancer [1,2]. The two primary types of IBD are Crohn’s Disease (CD) and Ulcerative Colitis (UC). CD affects the GI tract anywhere from mouth to anus with the frequent presentation of abdominal pain, fever, weight loss, and clinical signs of bowel obstruction or diarrhea [3]. UC on the other hand affects colon (large intestine) alone, starting from rectum and extending proximally through the entire colon. Inflammation in UC is restricted to the mucosal surface of the colon, manifesting as continuous areas of inflammation, ulceration, edema and hemorrhage [4]. The pathology of CD is characterized by the T helper (Th) 1 response, which is mediated by high levels of tumor necrosis factor (TNF)-α, interferon (IFN)-γ, and tissue-infiltrating Th17 cells. In contrast, the pathology of UC is characterized by the atypical Th2 response, mediated by high levels of Th17 cells [5,6].

## 2. Prevalence

The prevalence of IBD has gradually increased in recent years and varies according to geographical location including urban *vs*. rural areas [7,8]. In the United States alone, one to two million people have IBD while several million have it worldwide [7,8,9]. The prevalence of UC varies from 4.9 to 505 per 100,000 inhabitants in Europe, 4.9 to 168.3 per 100,000 inhabitants in Asia and the Middle East, and 37.5 to 248.6 per 100,000 inhabitants in North America. CD estimates range from 0.6 to 322 per 100,000 in Europe, 0.88 to 67.9 per 100,000 in Asia and the Middle East, and 16.7 to 318.5 per 100,000 in North America [7,8,9,10,11]. The prevalence of UC seems to increase with age [11,12,13]. In the last few decades, an increase in incidence occurred in industrialized zones which earlier had low incidence of IBD such as South Korea, China, India, Iran, Lebanon, Thailand, the French West Indies, North Africa and Japan, has been reported [10,14]. IBD is thus emerging as an important health problem worldwide. This condition mostly affects young people of both the sexes in the age group between 15 and 35 years [15]. Furthermore, IBD is associated with considerable healthcare costs [16,17]. Whereas childhood onset IBD represents only 10% to 25% of all IBD cases, genetic research of pediatric IBD has contributed new knowledge and revealed unsuspected pathways. A substantial proportion of patients with monogenic diseases present with very early onset intestinal inflammation (at less than 10 years of age) that is reminiscent of very early onset IBD. There is also considerable overlap with primary immune-deficiencies and very early onset IBD, a topic which has been reviewed recently [18].

## 3. Multi-Factorial Causes of IBD

**Figure 1:**
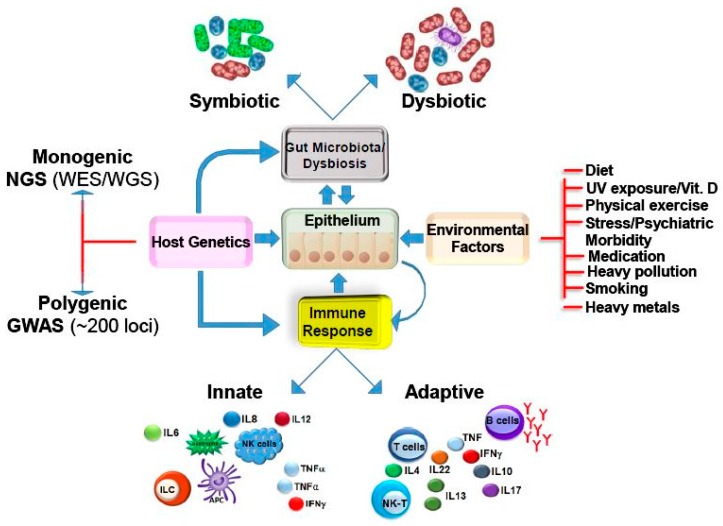
The cross-talk amongst the intestinal epithelium, gut microbiota, environmental factors and immune response along with host genetics dictates IBD pathogenesis. The intestinal epithelium is at the crossroad of IBD pathogenesis by coordinating the link amongst the factors implicated in the disease onset such as microbial flora, environmental factors, or the host immune response by directly interacting with those factors. Both the innate and adaptive immune responses show a disturbance in homeostasis. Flares of diseases have been associated with environmental factors, such as use of antibiotics and NSAIDs, stress and smoking. These factors or infections are thought to alter the barrier function of the epithelium, leading to loss of immune tolerance to intestinal antigens. The role of genetic factors is indicated by familial clustering of cases and higher incidence in monozygotic twins. Host genetics can itself influence the gut microbial composition or immune response to affect the disease pathogenesis.

Despite numerous studies, the actual causes of IBD are not known. However, the development and course of IBD may be affected by the complex interactions between genetic [19,20], environmental including breast feeding, diet, smoking, drugs [9] *etc*., and microbial factors [21], producing sustained inflammation supported by altered mucosal barrier and defects in immune system (Figure 1) [22]. The genetic basis of IBD was recognized early in clinical practice in view of the familial prevalence of IBD, concordance rates in twin pairs and ethnic differences in disease susceptibility [23,24,25,26,27]. Most identified and robustly replicated loci have been detected by means of genome-wide association studies (GWAS) [19,28,29]. GWAS have identified 163 loci related to the development of IBD [30], out of which 110 loci are shared between CD and UC, others are specifically associated with CD(30 loci) or UC (23 loci) [31]. These findings indicate that same mechanistic pathways and contribution occur in both the disease conditions. In CD patients, the alterations in innate immunity genes, such as NOD2 (also known as CARD 15), ATG16L1 (autophagy-related gene), and IRGM (immunity-related GTPase family), have been reported. In addition, multiple genes implicated in the IL-23 pathway (IL23R, IL12B, STAT3, JAK2 and TYK2) are associated with both UC and CD [32,33]. Association of immune related genes to IBD susceptibility and the development of intestinal inflammation in animal models with defective gastrointestinal immune response suggest that IBD may be caused by a dysregulated gastrointestinal immune response towards microbiota. Moreover, not all individuals with IBD-associated genetic variants develop the disease. Classic loss-of-function variants play only a disease initiation role in pathogenesis. Tobacco smoking has been consistently associated with the increased disease risk to CD but appears to be protective in UC [34]. Some studies suggest the role of diet in the etiology of IBD. Protein rich “Western” style diet has been shown to be associated with an increased risk for the development of CD, and possibly for the UC as well [35]. Antibiotics and non-steroidal anti-inflammatory agents (NSAIDs) are recognized as being capable of inducing or reactivating both CD and UC, and are thought to influence the progress of IBD by directly damaging the intestinal mucosa through the reduction of prostaglandin production [36]. Social stress has also been proposed to have a role in both diseases. In fact, mood components of perceived stress, such as depression, may play a strong role in mediating the deterioration of IBD [37]. Finally, while genetic contribution towards IBD pathogenesis has been enumerated, more recently, epigenetic factors have been shown to interact with the environment and genome and these factors can affect the development and progression of IBD. Thus, further investigations are required from all angles to explain the etiology of this disease [38]. Another related disorder, irritable bowel syndrome (IBS), is a disorder of the interaction between the brain and the GI tract, although abnormalities in the gut microbiota are implicated in inflammation and altered bowel function [39], Younger age, prolonged fever, anxiety, depression, and history of childhood physical and psychological abuse are often associated with the development of IBS after acute infectious gastroenteritis [40]. 

## 4. Microbiota in Health

The human body is colonized by a vast number of microorganisms representing the so-called normal microflora, the microbiota. The microbiota comprises mainly bacteria; however, viruses, fungi and protozoans live in a mutually beneficial relationship with the host. Microbiota colonizes the surface of the human body exposed to the external environment, including the skin, oral cavity, respiratory, urogenital and gastrointestinal tract. Of these, the gastrointestinal (GI) tract is the most densely colonized organ with about 100 trillion diverse microbes which is 10 times the number of all body cells [41] although recent studies refute this claim suggesting that there is a ratio of 1.3 bacteria to every one human cell [42]. The microbes of gut represent an ecosystem of the highest complexity [43] comprising over 1000 bacterial species and 150-fold more genes than found in the human genome [44,45]. The number and composition of microbiota varies in different regions of the GI tract with a relatively low number and few species residing in the stomach and upper small intestine. However, there is a diverse and dense population of microbiota in distal part of the small intestine and colon ranging up to 10^11^/g to 10^12^/g of luminal contents [46]. Metagenomic research that provides access to the functional gene composition of microbial communities, suggests that gut microbiota is mainly dominated by the Gram-negative Bacteroidetes (17%–60%) and Gram-positive Firmicutes (35%–80%) [45]. The other less prevalent phyla include *Actinobacteria*, *Proteobacteria* and *Euryarchaeota* [47,48]. The composition of the gut microbiota is dynamic and is influenced by a range of factors that include host genetics and immunity, the microbial species acquired at birth, antibiotic usage [49,50] and environmental factors such as diet [51,52,53,54].

In healthy individuals, gut microbiota lives symbiotically with the host and allows digestion of otherwise indigestible carbohydrates to produce short chain fatty acids (SCFA) to protect against epithelial injury, regulate fat metabolism, synthesize vitamins (e.g., vitamin K, vitamin B12 and folic acid) and essential amino acids, biotransform conjugated bile acids, cause intestinal motility, boost intestinal angiogenesis, and promote proper development of the immune system [55,56,57,58]. In addition, the gut microbiota resist the colonization of pathogenic bacteria and produce antimicrobial compounds. Thus, gut microbiota protect gut epithelial barrier from the harmful effects of pathogens, prevent bacterial overgrowth and reduce host susceptibility to enteric infections [59]. The diversity of the microbiome alters across body sites, between people, and with age and is diet-dependent, resulting in a series of unique habitats within and between individuals that are subject to temporal variation and variation between populations [60,61]. However, although inter-individual variability in microbial composition is amazingly diverse, recent meta-transcriptomic studies suggest that many of these microbial genes that differ between individuals may in fact be phenocopies and therefore capable of carrying out the same functions for the host [62]. The question as to what constitutes a healthy microbiome remains largely unanswered because of the uniqueness of the microbiome of each individual, especially at the species and strain level, although there are clearly communities at the family and class levels that have been identified as consistent with gut health [63].

## 5. Dysbiotic and/or Pathogenic Bacteria in IBD

Dysbiosis is defined as an increase in pathogenic bacteria concomitant with decreases in beneficial bacterial species [64]. The healthy host has a tolerance towards microbiota, and maintains immune homeostasis. Dysregulation of this homeostasis is a defining event in the development of IBD. Indeed, several studies conducted in patients and in animal models have clearly shown the central role of bacteria in the pathogenesis of IBD. Some of the most convincing pieces of evidence come from germ free mouse models, which develop chronic intestinal inflammation after colonization with commensal gut bacteria, but remain disease free in bacteria-free conditions, suggesting a primary role of non-pathogenic enteric bacteria in the pathogenesis of UC [65,66]. This led to the current theory of ‘‘no bacteria, no IBD’’ [67,68]. In addition, several findings suggest that the use of “beneficial bacteria” or probiotics can ameliorate IBD [69,70].

Recent metagenomic studies suggest that both quantity and composition of microbiota changes during IBD (Table 1). In general, an overall decrease in microbial diversity and stability of the intestinal microbiota has been observed in IBD patients [71]. On average, 25% fewer genes could be detected in the fecal samples of IBD patients compared with individuals not suffering from IBD [45]. These results infer that the microbiota of IBD patients has a lower functional diversity compared to healthy individuals. In comparison to healthy controls, the IBD patients have fewer bacteria with anti-inflammatory properties and/or more bacteria with pro-inflammatory properties.

The most well defined change that several metagenomic-based studies have noted in patients with IBD is the reduced abundance of the phyla *Firmicutes* [72,73,74,75]. Fecal microbiota analysis of CD patients show decreased presence of anti-inflammatory *F. prausnitzii, B. adolescentis, D. invisus* and an unknown species of *Clostridium cluster XIVa*, and an increased presence of potentially proinflammatory *R. gnavus* [76]. However there are contradictory reports regarding phylum *Bacteroidetes* wherein, some studies show reduced abundance during IBD [51,77,78,79] while others report increases in *Bacteroidetes* in IBD patients [80,81]. Likewise, most of the known pathogenic bacteria in human gastrointestinal disease belong to the phylum *Proteobacteria* [82]. Microbial diversity analysis has shown dual finding of decrease in *Firmicutes* associated with parallel increase in *Proteobacteria*, suggesting their key role in IBD [80,83,84,85,86]. While these data clearly suggest that dysbiosis may play an important role in the pathogenesis of IBD, it remains to be seen whether changes in phylogenetic composition are causative in the onset of IBD or simply a consequence of an altered gastrointestinal environment that affects the disease process.

Increased concentrations of *Escherichia coli* including pathogenic variants have been documented in ileal CD [87,88]. *E. coli* has been studied extensively in IBD patients and a new pathogenic group, namely adherent-invasive *E. coli* (*AIEC*) has been designated [89]. It has been reported that compared to healthy controls, the IBD patients have abnormal colonization of *AIEC* in ileal mucosa [90]. About 38% of patients with active ileal CD have *AIEC* while normal controls and patients with colonic CD contain very low percentage of this strain [84,90]. AIEC initiates chronic inflammation in susceptible hosts by altering the gut microbiota composition that gives it an inherently greater ability to activate innate immunity/pro-inflammatory gene expression. Similarly, Western diet induces changes in gut microbiota composition and alters host homeostasis to promote AIEC gut colonization in genetically susceptible mice. In humans, mucosal-associated *E. coli* are commonly found in inflamed tissues during IBD. While it is true that these bacteria often possess an adherent and invasive phenotype, they lack virulence-associated features of well-described intestinal *E. coli* pathogens, and are of diverse serology and phylotypes, making it difficult to correlate strain characteristics with the exacerbation of the disease. It is also true that AIEC-like isolates are more abundant in Crohn’s disease patients while the prevalence of *AIEC* is not high in UC patients [90]. Likewise, coinfection with *Mycobacterium avium* subsp. *paratuberculosis* (MAP) and AIEC is common and persistent in CD. However, high MAP and *E. coli* detection in cirrhotic patients with ascites suggests that colonization is, at least partially dependent on increased gut permeability. Since majority of the studies overwhelmingly and definitively support the role of MAP in at least 30%–50% of CD patients [91], facilitative mechanisms between a susceptible host and these two potential human pathogens may allow their implication in CD pathogenesis [92].

A second adherent, invasive proteobacterium, *Campylobacter concisus*, has also been associated with IBD [82,93,94,95]. *C. concisus* invasion affects membrane permeability and drives inflammation in host epithelial cells. Intestinal inflammation can also be caused by other enteric bacterial pathogens. *Clostridium difficile* toxin A for example, is associated with acute inflammation and fluid secretion. Toxin A can cause enterocyte apoptosis and inflammation in experimental models [96], and may have the ability to reactivate IBD [97]. *Bacteroides fragilis* is a normal colonic commensal bacterial species found in the majority of adults. One of its subset strains, termed enterotoxigenic *B. fragilis* (ETBF), secretes a pro-inflammatory zinc-dependent metalloprotease toxin that is associated with diarrheal illnesses in children and adults. ETBF is present in 19.3% of patients with clinically active IBD [77]. In animal studies, ETBF has been shown to cause colitis with severe inflammation and overproduction of interleukin-17 (IL-17), a central regulator of inflammation and autoimmunity [98].

## 6. Dietary Strategies Affecting the Microbiome, Metabolome and IBD

The gut is colonized by bacteria during birth and their composition is determined by the mode of delivery [54,99]. Gut microbiota becomes stable and adult like around 2–3 years of age [100] starting with the introduction of solid foods into the diet [61,101]. Several studies have explored the impact of diet on the newborn gut microbiota and have compared breastfeeding with formula feeding. A consistent finding has been the higher proportion of *Bifidobacteria* in breastfed infants as compared to formula-fed infants [102,103,104,105]. Several studies have examined the association between dietary patterns and the incidence of IBD [106,107]. It has been proposed that increased and refined carbohydrates and animal fat/protein and reductions in dietary fibers are major etiologic factors in the development of both UC and CD [108,109]. Consumption of high dietary intake of total fats, polyunsaturated fatty acids (PUFAs), omega-6 fatty acids, and meat are associated with an increased risk of CD and UC; high fiber and fruit intakes with a decreased CD risk; and high vegetable intake with a decreased UC risk [107]. In one study, children in Burkina Faso, a country with low incidence of IBD, fed with high-fiber, plant-based diet exhibited different gut microbial community than their European counterparts who consumed sugar, fat and protein rich diet [110]. The results were similar when the microbiota of healthy individuals from South America and South Asia were compared with healthy individuals from an industrialized country such as the United States [61]. These studies support the idea that the alteration of gut microbiota community structure through the consumption of agrarian *vs*. a “Westernized” diet may play a role in either reducing or increasing, respectively, the risk for the development of IBD. It is surprising, however, that there is little evidence to show that any specific dietary component acts as a risk factor for the development of UC or CD [111,112,113].

According to the existing literature, diet may serve as a symptomatic treatment for irritable bowel syndrome-like symptoms in IBD. Although the evidence is not substantial, enteral nutrition (EN) may be useful for maintaining remission in patients with quiescent Crohn’s disease. In pediatric patients with CD, EN reaches remission rates similar to steroids [114]. In adult patients however, meta-analyses have shown EN to be inferior to corticosteroids in adults with active Crohn’s disease while EN is not effective in UC [114]. A significant change occurs in the production of microbial metabolites after enteral feeding in both healthy volunteers and patients with CD. Many of those detected in CD are toxic and may feasibly lead to the immunological attack on the gut microbiota, which is characteristic of IBD. The reduction in the levels of such metabolites after enteral feeding may be the reason for its effectiveness in CD [115]. Exclusive enteral nutrition (EEN) refers to the exclusive use of liquid diet in an effort to induce remission in CD. Proposed mechanisms for the efficacy of EEN include alterations of the microbiota. A recent study used high throughput sequencing to determine changes in fecal microbiota before and after EEN in children with CD. Results showed decrease in number of operational taxonomic units after starting EEN, which corresponded with remission. In addition, recurrence of disease corresponded with increase in operational taxonomic units [116]. Other possible mechanisms include improved epithelial barrier function and anti-inflammatory effects [117].

A meta-analysis of pediatric studies showed remission rates with EEN that were equivalent to those of corticosteroids [118] and other studies have suggested greater rates of remission in ileal or ileo-colonic CD than in colonic phenotypes [119]. Due to the reduced palatability of EEN, in clinical practice food is typically slowly reintroduced after 8–12 weeks of EEN. In many cases, this leads to disease recurrence, however the period of remission allows for initiation of immunomodulators that may take weeks to become efficacious. Since gut dysbiosis is believed to play a role in the pathogenesis of IBD, fecal microbiota transplantation (FMT) is an effective strategy to restore intestinal microbial diversity and has been reported to have a potential therapeutic value in IBD. A recent study reported a holistic integrative therapy called “step-up FMT strategy,” which was beneficial in treating steroid-dependent IBD patients. This strategy consists of scheduled FMTs combined with steroids, anti-TNF-α antibody treatment or EN [120].

Total parenteral nutrition in IBD is not superior to steroids or EN. Despite the preference for enteral nutrition, some patients are unable to utilize their gut and therefore require parenteral nutrition (PN) although there are complications associated with the approach and mechanisms behind these complications are multifactorial and have yet to be fully elucidated. Recent studies utilizing both animal and human models have provided further information regarding parenteral nutrition’s deleterious effect on intestinal epithelial barrier function along with the complications associated with enterocyte deprivation. Parenteral nutrition has been a life-saving treatment in infants intolerant of enteral feedings. However, PN is associated with liver injury (PN Associated Liver Injury: PNALI) in a significant number of PN-dependent infants. Microbiome analysis in the PNALI mouse identified specific alterations within colonic microbiota associated with PNALI and further association of these communities with the lipid composition of the PN solution. Intestinal inflammation or soy oil-based PN infusion alone (in the absence of enteral feeds) caused shifts within the gut microbiota. However, the combination resulted in accumulation of a specific taxon, *Erysipelotrichaceae* (23.8% *vs*. 1.7% in saline infused controls), in PNALI mice. Moreover, PNALI was markedly attenuated by enteral antibiotic treatment, due partially to significant reduction of *Erysipelotrichaceae* (0.6%) and a Gram-negative constituent, the S24-7 lineage of Bacteroidetes. Importantly, removal of soy oil based-lipid emulsion from the PN solution resulted in significant reduction of *Erysipelotrichaceae* as well as attenuation of PNALI. Finally, addition of soy-derived plant sterol to fish oil-based PN restored *Erysipelotrichaceae* abundance and PNALI [121].

Animal disease models provide better evidence for the involvement of specific dietary components in the etiology of IBD. Using the genetically susceptible IL-10 deficient mouse model, Devkota *et al.* showed that feeding a Western-based diet rich in saturated milk fat elicited negative effects on intestinal health. This diet alters host production of the secondary bile acid taurocholate and provides an organic sulfur source for δ-proteobacteria, *B. wadsworthia* to bloom and thus increasing the incidence and severity of Th1-mediated spontaneous colitis [122,123]. It is quite evident that diet not only affects the composition and richness of the gut microbiota but also impacts the microbial metabolome by serving as a substrate to gut microbiota for the production of small molecules that impact host physiology [63].

Metabolomics is defined as a comprehensive and quantitative analysis of the small molecule metabolites synthesized by a biological system [124]. It is less invasive yet a robust and sensitive means of identifying metabolites produced by microbes and host cells in urine, serum, tissue or feces [125,126]. Metabolomics and metabolite profiling have been widely used to identify disease biomarkers. For example, the first microbiome studies sought to identify taxa that correlated with disease, physiological state, drug use, or dietary intake. However, not all exposures can alter the composition of the microbial community or its gene content; some can affect gene expression [127,128]. Humanized mice (created by transplanting human fecal microbiota into the mouse gut) have metabolomes distinct from those of conventionally raised mice [129]. This observation indicates that different gut microbes can produce changes in metabolites throughout their host.

Metabolomics has fundamentally and conceptually been divided into four major areas: target analysis, metabolite profiling, metabolomics, and metabolic fingerprinting [130]. While target analysis includes measurement of a small set of known metabolites, metabolite profiling analyses a larger set of compounds using GC-MS, including plants [131], microbes [132], urine [133], and plasma samples [134]. Metabolomics basically employs complementary methodologies including LC-MS/MS, GC-MS, and/or NMR to determine and quantify metabolites. Finally, during metabolic fingerprinting, a metabolic signature of the sample of interest is developed to screen for differences between the samples and once the metabolites are identified, the biological relevance of that compound can be determined that greatly reduces the analysis time.

Various metagenomic studies suggest that the metabolites derived from diverse microbial community can affect human health and disease [135] (Table 1). In a murine model of DSS-induced colitis, a total of 77 and 92 metabolites were detected in serum and colon tissue, respectively, and among the metabolites the compositions of TCA cycle intermediates and amino acids changed depending on the degree of colitis. Using a multiple classification analysis tool, partial least square discriminant analysis (PLS-DA), distinct clustering and clear separation of the groups was based on the degree of colitis. Furthermore, PLS-DA loading plots revealed that succinic acid, indole-3-acetic acid, glutamic acid, and glutamine were the main contributors to the separation of each stage of colitis. In addition, it was revealed that supplementation with glutamine, the level of which was significantly decreased in the acute phase of colonic inflammation, attenuated colitis induced by DSS [136].

In a human study published in 2014 [137], the metabolites that allowed to distinguish between the group of patients with active IBD and the group with IBD in remission were: N-acetylated compounds and phenylalanine which were up-regulated in serum, low-density lipoproteins and very low-density lipoproteins that decreased in the serum along with glycine that increased in urine and acetoacetate that exhibited a reduced levels in the urine. The significant differences in metabolomic profiles were also found between the group of patients with active IBD and healthy controls providing the PLS-DA models with a very good separation (*p* value < 0.001 for serum and 0.003 for urine). The metabolites with the strongest biomarkers included in this case: leucine, isoleucine, 3-hydroxybutyric acid, N-acetylated compounds, acetoacetate, glycine, phenylalanine and lactate that increased in serum, creatine, dimethyl sulfone, histidine, choline and its derivatives that decreased in serum as well as citrate, hippurate, trigonelline, taurine, succinate and 2-hydroxyisobutyrate that decreased in urine. No clear separation in PLS-DA models was found between CD and UC patients based on the analysis of serum and urine samples, although one metabolite (formate) in univariate statistical analysis was significantly lower in serum of patients with active CD, and two metabolites (alanine and N-acetylated compounds) were significantly higher in serum of patients with CD when comparing jointly patients in the remission and active phase of the diseases. Contrary to the results obtained from the serum samples, the analysis of urine samples allowed distinguishing patients with IBD in remission from healthy control subjects. The metabolites of importance included in this case up-regulated acetoacetate and down-regulated citrate, hippurate, taurine, succinate, glycine, alanine and formate.

A more recent study examined the metabolic activity in CD, UC or pouchitis patients and compared with healthy controls (HC) to determine whether eventual differences might be related to the pathogenesis of the disease. The number of metabolites identified in HC (54) was significantly higher than in patients with CD (44, *p* < 0.001), UC (47, *p* = 0.042) and pouchitis (43, *p* = 0.036). Multivariate discriminant analysis predicted HC, CD, UC and pouchitis group membership with high sensitivity and specificity. The levels of medium-chain fatty acids (MCFAs: pentanoate, hexanoate, heptanoate, octanoate and nonanoate), and of some protein fermentation metabolites, were significantly decreased in patients with CD, UC and pouchitis. Hexanoate levels were inversely correlated to disease activity in CD (correlation coefficient = −0.157, *p* = 0.046), whereas a significant positive correlation was found between styrene levels and disease activity in UC (correlation coefficient = 0.338, *p* = 0.001) [138].

Finally, effect of low fermentable oligosaccharides, disaccharides and monosaccharides and polyols (FODMAP) and high FODMAP diets on symptoms, the metabolome and the microbiome of patients with IBS was investigated. Thirty-seven patients (19 low FODMAP; 18 high FODMAP) completed the 3-week diet. The IBS symptom severity scoring (IBS-SSS) was reduced in the low FODMAP diet group (*p* < 0.001) but not the high FODMAP group. Lactulose breath test (LBTs) showed a minor decrease in H_2_ production in the low FODMAP compared with the high FODMAP group. Metabolic profiling of urine showed groups of patients with IBS differed significantly after the diet (*p* < 0.01), with three metabolites (histamine, p-hydroxybenzoic acid, azelaic acid) being primarily responsible for discrimination between the two groups. Histamine, a measure of immune activation, was reduced eightfold in the low FODMAP group (*p* < 0.05). Low FODMAP diet increased Actinobacteria richness and diversity, and high FODMAP diet decreased the relative abundance of bacteria involved in gas consumption [139].

The dietary components that escape digestion in the upper gastrointestinal tract provide most of the substrates for the intestinal microbiota. Fermentation of carbohydrates by the intestinal microbiota leads to the production of short chain fatty acids (SCFAs) such as butyrate, propionate, and acetate. Studies have shown that patients with inflammatory bowel diseases such as ulcerative colitis have fewer butyrate producing bacteria (e.g., *Roseburia hominis* and *Faecalibacterium prausnitzii*) in their intestine, resulting in lower levels of butyrate [140,141]. In addition to butyrate, propionate can potentiate *de novo* generation of regulatory T cells in the peripheral immune system. Modulation of butyrate- and propionate-producing microbes might therefore be used to treat inflammatory bowel diseases such as ulcerative colitis. Indeed, drug companies are now targeting receptors for these metabolites with small molecules [142]. Despite these advances however, the anti-inflammatory mechanisms of butyrate and other short-chain fatty acids remain poorly defined and clinicians continue to struggle with putting patients on low FODMAP (fermentable, oligo-, di-, monosaccharides, and polyols) diet that offers lower rates of abdominal pain, bloating, gas and diarrhea. Suffice to say, IBD patients need lot of nutritional advice since a credibility gap exists when it comes to diet and IBD. Studies of IBD patients have also shown that even when inflammation is in remission, the altered enteric nerves and abnormal microbiota can generate IBS-like symptoms. The efficacy of the low FODMAP diet as a treatment for bloating, flatulence, and abdominal discomfort has been demonstrated by randomized controlled trials. MRI studies, which can quantify intestinal volumes, have provided new insights into how FODMAPs cause symptoms [143].

## 7. Clinical Significance of Metabolomics

UC and CD are two distinct forms of IBD and are distinguished on the basis of variety of clinical, endoscopic, radiologic, serologic and pathologic evaluations. Unfortunately, there is a very thin line of distinction between these two diseases due to overlapping of etiological, clinical, and pathological features making it difficult to accurately diagnose the disease, especially in the pediatric age group. For tailored clinical management, it remains a challenge in 5%–20% cases to distinguish between UC and CD [144,145,146,147] which otherwise would lead to misclassification or repeated examinations [148]. The diagnosis of IBD using clinical, endoscopic, radiologic and histologic examination implicates that diagnosis is only possible at a relatively advanced stage of the disease. However, it would be useful for primary diagnosis, surveillance, and early detection of relapses to use less invasive yet more informative methods such as analysis of biomarkers from urine, serum, or feces. Fortunately some metabolomic biomarkers have been tested in clinical trials including C-reactive protein, fecal markers (lactoferrin, calprotectin, and PMN-elastase) and serological markers (antibodies against luminal antigens and anti-glycan antibodies) [149]. The spectrum of antibodies to different microbial antigens and autoantibodies associated with IBD is rapidly expanding. Most of these antibodies are associated with CD like anti-glycan antibodies: anti-Saccharomices cerevisiae (ASCA) and the recently described anti-laminaribioside (ALCA), anti-chitobioside (ACCA), anti-mannobioside (AMCA), anti-laminarin (anti-L) and anti-chitin (anti-C) antibodies; in addition to other antibodies that target microbial antigens: anti-outer membrane porin C (anti-OmpC), anti-Cbir1 flagellin *etc*. In addition, autoantibodies targeting the exocrine pancreas (PAB) were shown to be highly specific for CD [150]. Patients who are ASCA-positive have been shown to be more likely to have Crohn’s than UC, and more likely to have ileal disease than patients who are ASCA-negative. In addition, ASCA-positive patients may be more likely to undergo ileocecal resection [151]. Anti-glycan, anti-GP2 and anti-GM-CSF antibodies are especially associated with CD and seem to be correlated with complicated disease phenotypes even if results differ between studies. Although anti-glycan Ab and anti-GP2 Ab have low sensitivity in diagnosis of IBD, they could identify a small number of CD patients not detected by other tests such as ASCA. Anti-glycan Abs are associated with a progression to a more severe disease course and a higher risk for IBD-related surgery. Anti-GP2 Ab could particularly contribute to better stratify cases of pouchitis. Anti-GM-CSF Ab seems to be correlated with disease activity and could help predict relapses [143]. In contrast, UC has been associated with anti-neutrophil cytoplasmic autoantibodies (pANCA) and antibodies against goblet cells (GAB). Current evidence suggests that serologic panels of multiple antibodies are useful in differential diagnosis of CD *versus* UC and can be a valuable aid in stratifying patients according to disease phenotype and risk of complications [152].

To date, ^1^H NMR spectroscopy has been employed to characterize activity and severity of human IBD. Several studies have been performed on small and non-complex molecules, such as amino acids and related metabolites, on TCA cycle intermediates, and on metabolites involved in fatty acid and purine metabolism to compare between IBD patients and matched healthy subjects. Indeed there were differences in these metabolic profiles between IBD patients and healthy controls [153,154,155,156,157,158] as well as between the IBD subtypes [153,154,158]. Other common technologies to study metabolomics include gas chromatography-mass spectrometry (GC-MS) and Ion-cyclotron resonance-Fourier transform mass spectrometry (ICR-FT/MS) with ultrahigh mass resolution that can measure small but complex metabolites [159].

## 8. Conclusions

In conclusion, it is obvious that significant interdependence of the mucosal metabolome and microbiome exists suggesting that metagenomic composition is predictive to a reasonable degree of microbial community metabolite pools. Thus, studying the response of various organisms to different stresses and environments at the genetic, transcript, protein, and metabolite levels using different methods and comparing these results with those of other organisms will strengthen their integration into a systems biology framework. The finding that certain metabolites strongly correlate with microbial community structure suggests that it is worth investigating metabolites as direct mediators of microbial-associated disease activity and that metabolites may be a direct target for monitoring and therapeutically manipulating microbial community function in IBD and other intestinal diseases associated with dysbiosis.

## Figures and Tables

**Table 1:** Alterations in the microbiome and metabolome during IBD.

S. No	Increased	Decreased	Increased	Decreased
1	Phylum *proteobacteria* [78,81]	Phylum *Firmicutes* [70,71,72,160]	**Colon mucosal tissue** CD: glucose, glycerophosphorylcholine UC: arginine, glucose, glycerophosphorylcholine, lysine [144]	**Colon mucosal tissue** CD: alanine, choline, formate, glutamine/glutamate, isoleucine/leucine/valine, lactate, myoinositol, succinate UC: alanine, choline, formate, glutamine/glutamate, isoleucine/leucine/valine, lactate, myoinositol, succinate [144]
2	Adherent-invasive *E. coli* (AIEC) [87], *Campylobacter concisus* [80], *Clostridium difficil*) [92], *Bacteroides fragilis* [75], *Bacteroides vulgatus, Klebssiella pneumonie, fusobacterium varium* [161])	Butyrate producing bacteria e.g., *Roseburia hominis* and *Faecalibacterium* [136]	**Fecal matter** CD: alanine, glycerol, isoleucine, leucine, lysine, valine UC: glutamate, lysine [162]	**Fecal matter** CD: acetate, butyrate, methylamine, Trimethylamine UC: methylamine, trimethylamine [162]
3	*R. gnavus* [74]	Microbial diversity [69]	**Urine** CD: formate, glycine, glycolate, guanidoacetate, methylhistidine UC: citrate, glycine, glycolate, guanidoacetate, methylhistidine [158]	**Urine** CD: 4-cresol sulfate, citrate, hippurate UC: hippurate, trimethyllysine [158]
4	*CD:Mycobacterium avium paratuberculosis* (MAP) [72]	Microbial genes in feces [43]	ND	SCFA synthesis [136]
5	Enterotoxigenic *B. fragilis* (ETBF) [98]	Decreased presence of anti-inflammatory *F. prausnitzii, B. adolescentis, D. invisus* [74]	ND	Amino acid biosynthesis [147]

ND = No description.
